# Correction: A consensus molecular subtypes classification strategy for clinical colorectal cancer tissues

**DOI:** 10.26508/lsa.202402889

**Published:** 2024-06-27

**Authors:** Tim R de Back, Tan Wu, Pascale JM Schafrat, Sanne ten Hoorn, Miaomiao Tan, Lingli He, Sander R van Hooff, Jan Koster, Lisanne E Nijman, Geraldine R Vink, Inès J Beumer, Clara C Elbers, Kristiaan J Lenos, Dirkje W Sommeijer, Xin Wang, Louis Vermeulen

**Affiliations:** 1 Cancer Center Amsterdam, Laboratory for Experimental Oncology and Radiobiology, Center for Experimental and Molecular Medicine, Amsterdam, Netherlands; 2 Amsterdam Gastroenterology Endocrinology Metabolism, Laboratory for Experimental Oncology and Radiobiology, Center for Experimental and Molecular Medicine, Amsterdam, Netherlands; 3 Oncode Institute , Amsterdam, Netherlands; 4 Key Laboratory of Genomic and Precision Medicine, Beijing Institute of Genomics, Chinese Academy of Sciences and China National Center for Bioinformation, Beijing, China; 5 Department of Surgery, The Chinese University of Hong Kong, Hong Kong SAR, China; 6 Amsterdam UMC Location Vrije Universiteit Amsterdam, Department of Medical Oncology, Amsterdam, Netherlands; 7 Institute of Translational Medicine, Zhejiang Shuren University, Hangzhou, China; 8 Department of Medical Oncology, University Medical Center Utrecht, Utrecht University, Utrecht, Netherlands; 9 Department of Research and Development, Netherlands Comprehensive Cancer Organisation, Utrecht, Netherlands; 10 GenomeScan B.V., Leiden, Netherlands; 11 Flevohospital, Department of Internal Medicine, Almere, Netherlands; 12 Li Ka Shing Institute of Health Sciences, The Chinese University of Hong Kong, Hong Kong SAR, China; 13 Shenzhen Research Institute, The Chinese University of Hong Kong, Shenzhen, China

## Abstract

This work presents a novel consensus molecular subtype (CMS) classifier for colorectal cancer (CRC), optimized for RNA-sequencing data stemming from degraded RNA of clinical formalin-fixed paraffin-embedded (FFPE) tissue samples (the CMSFFPE classifier).

Article: de Back TR, Wu T, Schafrat PJ, ten Hoorn S, Tan M, He L, van Hooff SR, Koster J, Nijman LE, Vink GR, Beumer IJ, Elbers CC, Lenos KJ, Sommeijer DW, Wang X, Vermeulen L (2024 May 23) A consensus molecular subtypes classification strategy for clinical colorectal cancer tissues. Life Sci Alliance 7(8): e202402730. doi: 10.26508/lsa.202402730. PMID: 38782602.

## Results Section

**-** Table 1: Column “Public ID” of the last row “FFPE-RNA application cohort” reads NA.

**○** Should read: GSE267010.

**-** Title of Figure 3: Mutations characteristics of the CMSs in the FFPE application cohort.

**○** Should read: Molecular characteristics of the CMSs in the FFPE application cohort.

**-** Paragraph “Molecular characteristics of the CMSs in the FFPE-RNA application cohort,” third sentence, reads: Next, we assessed gene set enrichment across the CMSs and could recover all previously reported iCMS features (Fig 3B).

**○** Should read: Next, we assessed gene set enrichment across the CMSs and could recover all previously reported CMS features (Fig 3B).

## Discussion Section

**-** Fourth paragraph, last sentence, reads: “Hence, the observed OS patterns appear as intrinsic feature of the selected patient group and further strengthen the robustness of the CMSFFPE classifier.”

**○** Should read: “Hence, the observed OS patterns appear as intrinsic features of the selected patient group and further strengthen the robustness of the CMSFFPE classifier.”

## Materials and Methods Section

**-** Paragraph “RNA-sequencing and targeted exome sequencing,” third paragraph, first sentence, reads: “The TSO500 High-Throughput assay was performed on genomic DNA of the FFPE-RNA application cohort as instructed by IlluminaPaired-end …”

**○** Should read: “The TSO500 High-Throughput assay was performed on genomic DNA of the FFPE-RNA application cohort as instructed by Illumina. Paired-end …”

## Supplementary Material

Reviewer comments

