# Supplementary material for: A consensus molecular subtypes classification strategy for clinical colorectal cancer tissues
Source: Life Sci Alliance. 2024 May 23;7(8):e202402730. doi: 10.26508/lsa.202402730 (PMC11116811; doi:10.26508/lsa.202402730)
Supplement: Supplementary file 1 [file LSA-2024-02730_TableS1.docx]

| **Table S1.** Sample information for the low-quality discovery set. | | | | |
| --- | --- | --- | --- | --- |
| FFPE Sample ID | Matched FF Sample ID (GSE33113) | Total reads | Unique alignment | TIN (median) |
| 7069-02-001-001 | GSM820049 | 21500937 | 0.59% | 0 |
| 7069-02-001-002 | GSM820051 | 36212724 | 4.90% | 13.08 |
| 7069-02-001-003 | GSM820066 | 30272571 | 4.42% | 8.63 |
| 7069-02-001-004 | GSM820067 | 31399437 | 1.10% | 12.29 |
| 7069-02-001-005 | GSM820068 | 39920734 | 0.53% | 2.45 |
| 7069-02-001-006 | GSM820085 | 35174114 | 0.55% | 6.15 |
| 7069-02-001-007 | GSM820089 | 35328608 | 4.39% | 13.45 |
| 7069-02-001-008 | GSM820058 | 24502923 | 1.13% | 28.15 |
| 7069-02-001-009 | GSM820061 | 30341767 | 2.86% | 14.57 |
| 7069-02-001-010 | GSM820121 | 47754467 | 5.38% | 13.14 |
| 7069-02-001-011 | GSM820125 | 28305609 | 4.31% | 12.93 |
| 7069-02-001-012 | GSM820127 | 31850545 | 33.84% | 17.77 |
| 7069-02-001-013 | GSM820133 | 19211245 | 14.05% | 13.32 |
| 7069-02-001-014 | GSM820135 | 31882209 | 21.35% | 13.95 |
| 7069-02-001-019 | GSM820070 | 22386582 | 2.12% | 3.71 |
| 7069-02-001-020 | GSM820103 | 20229688 | 2.11% | 10.21 |
| 7069-02-001-021 | GSM820097 | 23665604 | 0.92% | 3.73 |
| 7069-02-001-022 | GSM820120 | 38779014 | 20.99% | 18.90 |
| 7069-02-001-033 | GSM820068 | 19711726 | 0.87% | 4.24 |
| 7069-02-001-034 | GSM820096 | 22770069 | 19.33% | 15.95 |
| 7069-02-001-035 | GSM820125 | 18607272 | 7.79% | 14.94 |
| 7069-02-001-036 | GSM820127 | 16013913 | 1.62% | 10.61 |
| Median value | - | 29289090 | 3.59% | 13.01 |
