# Supplementary material for: A consensus molecular subtypes classification strategy for clinical colorectal cancer tissues
Source: Life Sci Alliance. 2024 May 23;7(8):e202402730. doi: 10.26508/lsa.202402730 (PMC11116811; doi:10.26508/lsa.202402730)
Supplement: Supplementary file 2 [file LSA-2024-02730_TableS2.docx]

| **Table S2.** Sample information for the moderate-quality discovery set. | | | | |
| --- | --- | --- | --- | --- |
| FFPE Sample ID | Matched FF Sample ID (GSE33113) | Total reads | Unique alignment | TIN (median) |
| COL011 | GSM820058 | 54M | 72.48% | 26.94 |
| COL014 | GSM820061 | 36M | 74.57% | 28.37 |
| COL020 | GSM820067 | 10M | 49.70% | 8.40 |
| COL047 | GSM820085 | 4M | 24.91% | 2.62 |
| COL051_1 | GSM820089 | 40M | 67.89% | 36.81 |
| COL051_2 | GSM820089 | 14M | 68.41% | 33.59 |
| COL084 | GSM820121 | 3M | 55.13% | 13.37 |
| COL090 | GSM820125 | 8M | 24.49% | 1.75 |
| COL092 | GSM820127 | 25M | 71.74% | 37.11 |
| COL098_1 | GSM820133 | 30M | 75.18% | 30.78 |
| COL098_2 | GSM820133 | 32M | 75.22% | 32.97 |
| COL100 | GSM820135 | 7M | 49.15% | 14.42 |
| Median value | - | 19.5M | 68.15% | 27.66 |
