# Supplementary material for: A consensus molecular subtypes classification strategy for clinical colorectal cancer tissues
Source: Life Sci Alliance. 2024 May 23;7(8):e202402730. doi: 10.26508/lsa.202402730 (PMC11116811; doi:10.26508/lsa.202402730)
Supplement: Supplementary file 3 [file LSA-2024-02730_TableS3.docx]

| **Table S3.** Sample information for the FFPE-RNA application cohort. | | | | |
| --- | --- | --- | --- | --- |
| FFPE Sample ID | Matched FF Sample ID | Total reads | Unique alignment | TIN (median) |
| 104613-001-003 | - | 120038601 | 41.75% | 67.35 |
| 104613-001-004 | - | 131423373 | 19.39% | 51.41 |
| 104613-001-006 | - | 136656448 | 26.33% | 58.56 |
| 104792-001-001 | - | 53735601 | 7.11% | 3.48 |
| 104792-001-002 | - | 141674280 | 24.80% | 49.89 |
| 104792-001-003 | - | 209630023 | 13.21% | 45.26 |
| 104792-001-004 | - | 135541544 | 11.06% | 19.40 |
| 104792-001-005 | - | 165051714 | 21.54% | 57.53 |
| 104792-001-006 | - | 164704071 | 9.18% | 29.50 |
| 104792-001-007 | - | 148723920 | 47.63% | 62.57 |
| 104792-001-008 | - | 78940799 | 40.86% | 59.93 |
| 104792-001-009 | - | 81062285 | 14.33% | 47.62 |
| 104792-001-011 | - | 73519926 | 19.84% | 56.81 |
| 104792-001-013 | - | 86313646 | 26.21% | 61.78 |
| 104792-001-014 | - | 79442734 | 24.98% | 40.40 |
| 104792-001-015 | - | 77945352 | 29.75% | 53.90 |
| 104792-001-016 | - | 86903696 | 29.58% | 58.11 |
| 104792-001-017 | - | 48496034 | 6.20% | 4.78 |
| 104792-001-018 | - | 117259084 | 44.87% | 67.10 |
| 104792-001-019 | - | 102330603 | 57.40% | 63.60 |
| 104792-001-020 | - | 104445813 | 30.14% | 56.37 |
| 104792-001-021 | - | 56729738 | 8.38% | 6.59 |
| 104792-001-022 | - | 89740030 | 25.72% | 61.67 |
| 104792-001-024 | - | 54947690 | 6.60% | 5.61 |
| 104792-001-025 | - | 36589376 | 5.35% | 4.37 |
| 104792-001-026 | - | 65860595 | 7.22% | 8.09 |
| 104792-001-027 | - | 91464765 | 31.67% | 60.54 |
| 104792-001-028 | - | 70987362 | 11.29% | 35.49 |
| 104792-001-030 | - | 44668982 | 7.54% | 3.89 |
| 104792-001-033 | - | 84575334 | 11.24% | 31.65 |
| 104792-001-034 | - | 52560885 | 7.11% | 4.41 |
| 104792-001-035 | - | 93582026 | 25.23% | 55.64 |
| 104792-001-036 | - | 62871509 | 8.00% | 8.45 |
| 104792-001-037 | - | 95715574 | 41.44% | 63.58 |
| 104792-001-039 | - | 99696085 | 15.00% | 38.22 |
| 104792-001-040 | - | 73225705 | 12.42% | 41.92 |
| 104792-001-041 | - | 36546059 | 5.04% | 3.25 |
| 104792-001-042 | - | 78931865 | 11.77% | 38.17 |
| 104792-001-043 | - | 88824324 | 24.28% | 49.21 |
| 104792-001-044 | - | 91487731 | 33.64% | 63.76 |
| 104792-001-045 | - | 41961801 | 7.12% | 3.01 |
| 104792-001-046 | - | 49953753 | 6.93% | 4.76 |
| 104792-001-047 | - | 97614991 | 41.79% | 68.19 |
| 104792-001-048 | - | 98625669 | 16.64% | 47.87 |
| 104792-001-049 | - | 119942320 | 39.01% | 68.42 |
| 104792-001-051 | - | 120370403 | 53.65% | 72.34 |
| 104792-001-052 | - | 110731640 | 44.71% | 70.51 |
| 104792-001-053 | - | 94707324 | 45.56% | 71.33 |
| 104792-001-054 | - | 64829890 | 14.45% | 18.59 |
| 104792-001-055 | - | 89345903 | 23.72% | 60.77 |
| 104792-001-056 | - | 73992829 | 14.04% | 23.21 |
| 104792-001-057 | - | 77143692 | 12.63% | 42.88 |
| 104792-001-058 | - | 114722768 | 51.51% | 72.08 |
| 104792-001-059 | - | 87433878 | 34.34% | 67.17 |
| 104792-001-061 | - | 103591967 | 43.09% | 69.84 |
| 104792-001-062 | - | 92549656 | 38.48% | 68.39 |
| 104792-001-063 | - | 66078124 | 8.00% | 9.30 |
| 104792-001-064 | - | 105314076 | 54.44% | 69.43 |
| 104792-001-065 | - | 103123489 | 49.55% | 70.22 |
| 104792-001-066 | - | 122727217 | 38.52% | 67.88 |
| 104792-001-067 | - | 83047755 | 34.60% | 64.01 |
| 104792-001-068 | - | 87791659 | 29.35% | 64.33 |
| 104792-001-069 | - | 93198966 | 36.85% | 67.57 |
| 104792-001-070 | - | 92934392 | 39.70% | 68.53 |
| 104792-001-071 | - | 94060098 | 43.70% | 67.41 |
| 104792-001-072 | - | 95982349 | 55.38% | 70.85 |
| 104792-001-073 | - | 91930674 | 34.25% | 67.42 |
| 104792-001-074 | - | 183662839 | 55.75% | 71.09 |
| 104792-001-075 | - | 57182151 | 11.45% | 12.29 |
| 104792-001-076 | - | 94065292 | 39.68% | 64.58 |
| 104792-001-077 | - | 90323377 | 41.65% | 65.84 |
| 104792-001-080 | - | 108387101 | 60.05% | 69.92 |
| 104792-001-082 | - | 89768543 | 46.70% | 67.50 |
| 104792-001-084 | - | 41658282 | 5.71% | 4.27 |
| 104792-001-085 | - | 86293359 | 28.26% | 62.37 |
| 104792-001-086 | - | 64606967 | 7.51% | 7.92 |
| 104792-001-087 | - | 60848434 | 7.23% | 10.22 |
| 104792-001-088 | - | 94475023 | 45.53% | 68.49 |
| 104792-001-089 | - | 49482398 | 7.31% | 6.01 |
| 104792-001-090 | - | 81070044 | 18.13% | 38.49 |
| 104792-001-091 | - | 97815155 | 32.16% | 64.26 |
| 104792-001-092 | - | 61066822 | 7.33% | 5.83 |
| 104792-001-093 | - | 77871229 | 12.98% | 28.07 |
| 104792-001-094 | - | 90575538 | 34.13% | 65.30 |
| 104792-001-095 | - | 95526683 | 43.37% | 69.22 |
| 104792-001-096 | - | 45909247 | 5.49% | 4.11 |
| 104792-002-001-002 | - | 86202802 | 39.22% | 67.86 |
| 104792-002-001-003 | - | 90042441 | 27.99% | 64.77 |
| 104792-002-001-004 | - | 92103296 | 49.95% | 74.63 |
| 104792-002-001-005 | - | 66966402 | 9.08% | 13.55 |
| 104792-002-001-006 | - | 105936236 | 59.43% | 63.28 |
| 104792-002-001-007 | - | 90222087 | 43.67% | 57.83 |
| 104792-002-001-008 | - | 95940188 | 24.24% | 64.03 |
| 104792-002-001-009 | - | 95644295 | 46.36% | 69.80 |
| 104792-002-001-010 | - | 96003147 | 39.37% | 70.22 |
| 104792-002-001-011 | - | 89191930 | 9.68% | 19.43 |
| 104792-002-001-012 | - | 84981406 | 18.38% | 28.83 |
| 104792-002-001-013 | - | 86508612 | 8.08% | 11.48 |
| 104792-002-001-014 | - | 92254149 | 51.69% | 72.53 |
| 104792-002-001-016 | - | 102114137 | 50.51% | 73.03 |
| 104792-002-001-017 | - | 69144291 | 9.81% | 4.51 |
| 104792-002-001-018 | - | 80694125 | 10.65% | 23.26 |
| 104792-003-001-001 | - | 101192092 | 24.33% | 59.64 |
| 104792-003-001-002 | - | 101377582 | 39.86% | 67.47 |
| Median value | - | 91464765 | 25.97% | 58.34 |
