# Supplementary material for: A consensus molecular subtypes classification strategy for clinical colorectal cancer tissues
Source: Life Sci Alliance. 2024 May 23;7(8):e202402730. doi: 10.26508/lsa.202402730 (PMC11116811; doi:10.26508/lsa.202402730)
Supplement: Supplementary file 4 [file LSA-2024-02730_TableS4.docx]

| **Table S4.** Genetic driver events per CMS in the FFPE-RNA application cohort. | | | | | |
| --- | --- | --- | --- | --- | --- |
| Gene(s) | CMS1  n=4 (%) | CMS2  n=55 (%) | CMS3  n=10 (%) | CMS4  n=31 (%) | *P*-value* |
| TP53 | 2 (50.0) | 49 (89.1) | 6 (60.0) | 18 (58.1) | 0.002 |
| APC | 1 (25.0) | 35 (63.6) | 6 (60.0) | 15 (48.4) | 0.312 |
| TP53 and APC | 1 (25.0) | 32 (58.2) | 4 (40.0) | 13 (41.9) | 0.321 |
| EGFR | 0 (0.0) | 12 (21.8) | 1 (10.0) | 1 (3.2) | 0.085 |
| ERBB2 | 1 (25.0) | 7 (12.7) | 0 (0.0) | 2 (6.5) | 0.350 |
| SOX9 | 0 (0.0) | 5 (9.1) | 0 (0.0) | 1 (3.2) | 0.666 |
| SMAD4 | 0 (0.0) | 4 (7.3) | 1 (10.0) | 1 (3.2) | 0.575 |
| ERBB3 | 0 (0.0) | 1 (1.8) | 0 (0.0) | 2 (6.5) | 0.553 |
| PIK3CA | 1 (25.0) | 5 (9.1) | 1 (10.0) | 2 (6.5) | 0.540 |
| NOTCH1 | 0 (0.0) | 2 (3.6) | 0 (0.0) | 2 (6.5) | 0.793 |
| TCF7L2 | 1 (25.0) | 2 (3.6) | 1 (10.0) | 1 (3.2) | 0.177 |
| ARID1A | 0 (0.0) | 3 (5.5) | 0 (0.0) | 0 (0.0) | 0.715 |
| CTNNB1 | 1 (25.0) | 1 (1.8) | 1 (10.0) | 0 (0.0) | 0.047 |
| **P*-values are calculated with the Chi-square test and the Fisher’s Exact test | | | | | |
