# Supplementary material for: A consensus molecular subtypes classification strategy for clinical colorectal cancer tissues
Source: Life Sci Alliance. 2024 May 23;7(8):e202402730. doi: 10.26508/lsa.202402730 (PMC11116811; doi:10.26508/lsa.202402730)
Supplement: Supplementary file 5 [file LSA-2024-02730_TableS5.docx]

| **Table S5.** Univariate cox regression analyses for overall survival. | | |
| --- | --- | --- |
| Variable | HR (95% CI) | *P*-value |
| Sex (missing = 0)  Male  Female (ref) | 1.60 (0.92-2.79)  1 | 0.097 |
| Age at diagnosis (missing = 0)  <51  51-60  61-70  >70 (ref) | 0.71 (0.33–1.53)  0.72 (0.35-1.50)  0.59 (0.28-1.24)  1 | 0.580 |
| Stage at diagnosis (missing = 1)  1  2  3  4 (ref) | 0.50 (0.07-3.61)  1.00 (0.24-4.11)  1.03 (0.47-2.27)  1 | 0.922 |
| Surgery of primary (missing = 0)  No  Yes (ref) | 3.74 (2.27-6.16)  1 | <0.001 |
| Anti-EGFR treatment (missing = 0)  No  Yes (ref) | 0.62 (0.37-1.05)  1 | 0.073 |
| Lines of systemic treatment (missing = 0)  0-1 (ref)  2  3  >3 | 1  2.54 (1.33-4.85)  2.69 (1.31-5.52)  2.91 (1.45-5.83) | 0.007 |
| CMS (missing = 0)  1  2 (ref)  3  4 | 1.05 (0.61-1.82)  1  0.89 (0.21-3.84)  1.70 (0.74-3.90) | 0.612 |
| Number of organs with meta’s (missing = 0)  0-1 (ref)  2  3  >3 | 1  1.48 (0.76-2.88)  2.46 (1.27-4.78)  2.35 (1.24-4.45) | 0.019 |
| Sidedness (missing = 0)  Unknown  Right-sided  Left-sided (ref) | 0.68 (0.09-4.93)  1.03 (0.54-1.96)  1 | 0.924 |
