# Supplementary material for: A consensus molecular subtypes classification strategy for clinical colorectal cancer tissues
Source: Life Sci Alliance. 2024 May 23;7(8):e202402730. doi: 10.26508/lsa.202402730 (PMC11116811; doi:10.26508/lsa.202402730)
Supplement: Supplementary file 6 [file LSA-2024-02730_TableS6.docx]

| **Table S6.** Genetic driver events per anti-EGFR responder group in the FFPE-RNA application cohort. | | | |
| --- | --- | --- | --- |
| Gene(s) | Non-responders  n=22 (%) | Responders  n=26 (%) | *P*-value* |
| TP53 | 14 (63.6) | 25 (96.2) | 0.017 |
| APC | 11 (50.0) | 19 (73.1) | 0.100 |
| TP53 and APC | 7 (31.8) | 19 (73.1) | 0.004 |
| EGFR | 3 (13.6) | 2 (7.7) | 0.649 |
| ERBB2 | 2 (9.1) | 2 (7.7) | 1 |
| SOX9 | 1 (4.5) | 2 (7.7) | 1 |
| SMAD4 | 1 (4.5) | 2 (7.7) | 1 |
| ERBB3 | 2 (9.1) | 1 (3.8) | 0.587 |
| PIK3CA | 2 (9.1) | 0 (0.0) | 0.205 |
| NOTCH1 | 1 (4.5) | 1 (3.8) | 1 |
| TCF7L2 | 0 (0.0) | 2 (7.7) | 0.493 |
| ARID1A | 0 (0.0) | 1 (3.8) | 1 |
| CTNNB1 | 0 (0.0) | 1 (3.8) | 1 |
| **P*-values are calculated with the Chi-square test and the Fisher’s Exact test | | | |
