# Supplementary material for: A consensus molecular subtypes classification strategy for clinical colorectal cancer tissues
Source: Life Sci Alliance. 2024 May 23;7(8):e202402730. doi: 10.26508/lsa.202402730 (PMC11116811; doi:10.26508/lsa.202402730)
Supplement: Supplementary file 7 [file LSA-2024-02730_TableS7.docx]

| **Table S7.** Univariable logistic regression analyses for overall response rate anti-EGFR therapy. | | |
| --- | --- | --- |
| Variable | OR (95% CI) | *P*-value |
| CMS (missing = 0)  2&3 (ref)  1&4 | 1  0.37 (0.11-1.23) | 0.104 |
| Stage at diagnosis (missing = 1)  1  2  3  4 (ref) | 0.79 (0.05-13.69)  0.00 (0.00-NA)  0.99 (0.23-4.33)  1 | 0.999 |
| Age at diagnosis (missing = 0)  <51  51-60  61-70  >70 (ref) | 0.53 (0.09-3.31)  0.64 (0.10-4.11)  1.49 (0.30-7.39)  1 | 0.560 |
| Sex (missing = 0)  Male  Female (ref) | 0.66 (0.18-2.43)  1 | 0.533 |
| Surgery of primary (missing = 0)  No  Yes (ref) | 0.95 (0.28-3.24)  1 | 0.938 |
| Sidedness (missing = 1)  Right-sided  Left-sided (ref) | 0.23 (0.04-1.30)  1 | 0.096 |
| Line of anti-EGFR treatment (missing = 0)  1 (ref)  2  3 of meer | 1  1.13 (0.22-5.86)  0.24 (0.04-1.51) | 0.088 |
| Number of distant metastases (missing = 0)  0-1 (ref)  2  3  >3 | 1  2.44 (0.41-14.75)  0.25 (0.04-1.56)  0.57 (0.11-3.04) | 0.095 |
| TP53 mutation (missing = 2)  No  Yes (ref) | 0.09 (0.01-0.77)  1 | 0.028 |
| APC mutation (missing = 2)  No  Yes (ref) | 0.37 (0.11-1.23)  1 | 0.104 |
| APC and TP53 mutation (missing = 2)  No  Yes (ref) | 0.17 (0.05-0.60)  1 | 0.006 |
