# Supplementary material for: Correction: A consensus molecular subtypes classification strategy for clinical colorectal cancer tissues
Source: Life Sci Alliance. 2024 Jun 27;7(9):e202402889. doi: 10.26508/lsa.202402889 (PMC11211997; doi:10.26508/lsa.202402889)
Supplement: Reviewer comments [file LSA-2024-02889_review_history.pdf]

Life Science Alliance

Correction: A consensus molecular subtypes classification strategy for clinical colorectal cancer tissues

Tim de Back, Tan Wu, Pascale Schafrat, Sanne ten Hoorn, Miaomiao Tan, Lingli He, Sander van Hooff, Jan Koster, Lisanne Nijman, Geraldine Vink, Ines Beumer, Clara Elbers, Kristiaan Lenos, Dirkje Sommeijer, Xin Wang, and Louis Vermeulen
DOI: https://doi.org/10.26508/lsa.202402889

Corresponding author(s): Louis Vermeulen, Amsterdam University Medical Centers

**Review Timeline:**	Submission Date:	2024-06-18
	Accepted:	2024-06-21

Scientific Editor: Eric Sawey, PhD

Transaction Report:

(Note: With the exception of the correction of typographical or spelling errors that could be a source of ambiguity, letters and reports are not edited. The original formatting of letters and referee reports may not be reflected in this compilation.)

June 21, 2024

RE: Life Science Alliance Manuscript #LSA-2024-02889

Prof. Louis Vermeulen
Amsterdam University Medical Centers
Meibergdreef 9
Amsterdam, Noord-Holland 1105 AZ
Netherlands

Dear Dr. Vermeulen,

Thank you for submitting your Correction entitled "Correction: A consensus molecular subtypes classification strategy for clinical colorectal cancer tissues".

IMPORTANT: If you will be unreachable at any time, please provide us with the email address of an alternate author. Failure to respond to routine queries may lead to unavoidable delays in publication.

You can contact the journal office with any questions, contact@life-science-alliance.org

Sincerely,

Eric Sawey, PhD
Executive Editor
Life Science Alliance
http://www.lsajournal.org

June 21, 2024

RE: Life Science Alliance Manuscript #LSA-2024-02889

Prof. Louis Vermeulen
Amsterdam University Medical Centers
Meibergdreef 9
Amsterdam, Noord-Holland 1105 AZ
Netherlands

Dear Dr. Vermeulen,

Thank you for submitting your Correction entitled "Correction: A consensus molecular subtypes classification strategy for clinical colorectal cancer tissues".

IMPORTANT: If you will be unreachable at any time, please provide us with the email address of an alternate author. Failure to respond to routine queries may lead to unavoidable delays in publication.

You can contact the journal office with any questions, contact@life-science-alliance.org

Sincerely,

Eric Sawey, PhD
Executive Editor
Life Science Alliance
http://www.lsajournal.org
